# Rare giant retroperitoneal melanotic schwannoma: a case report and literature review

**DOI:** 10.3389/fonc.2024.1448112

**Published:** 2024-08-29

**Authors:** Pan Chen, Junfeng Cheng, Lin Zhang

**Affiliations:** ^1^ Department of Gynecology, Jinhua Municipal Central Hospital, Jinhua, Zhejiang, China; ^2^ Department of Hepatopancreatobiliary Surgery, Jinhua Municipal Central Hospital, Jinhua, Zhejiang, China

**Keywords:** melanotic schwannoma(MS), retroperitoneal schwannoma, malignant melanotic nerve sheath tumor(MMNST), rare giant tumor, surgical resection

## Abstract

**Background:**

Melanotic schwannoma (MS), a rare variant of peripheral nerve sheath tumor, is especially infrequent when originating from the peritoneum. Its definitive diagnosis relies on postoperative histopathological examination and immunohistochemical analysis, while preoperative diagnosis is difficult.

**Case presentation:**

In the present study, we reported a rare case of giant MS in the retroperitoneum, which was previously misdiagnosed before surgery. However, intraoperative exploration revealed it was retroperitoneal tumor. The tumor had invaded the abdominal aorta and bilateral common illiac artery walls. A surgical resection was subsequently executed, and postoperative histopathological examination confirmed it as a MS.

**Conclusion:**

The incidence of peritoneal MS is extremely rare, and surgical resection remains the preferred treatment modality. Given the absence of established guidelines for postoperative adjuvant therapy, long-term follow-up becomes imperative to accumulate valuable clinical expertise.

## Introduction

MS, an exceedingly rare nerve sheath tumor, was first described and reported by Miler in 1932 as a malignant melanoma originating from the thoracic sympathetic ganglion (1). The malignant potential of MS remains uncertain, comprising 1% of all nerve sheath tumors. To date, around 300 cases have been reported in diverse anatomical locations, particularly affecting the cervical and thoracic spine. Notably, one of the rare location for MS is the retroperitoneum, where only nine cases have been reported. This study presents a rare case of retroperitoneal MS, which is a challenge in preoperative diagnosis and the potential for misdiagnosis. The case report offers valuable insights from a clinical perspective.

## Case presentation

**Figure 1:**
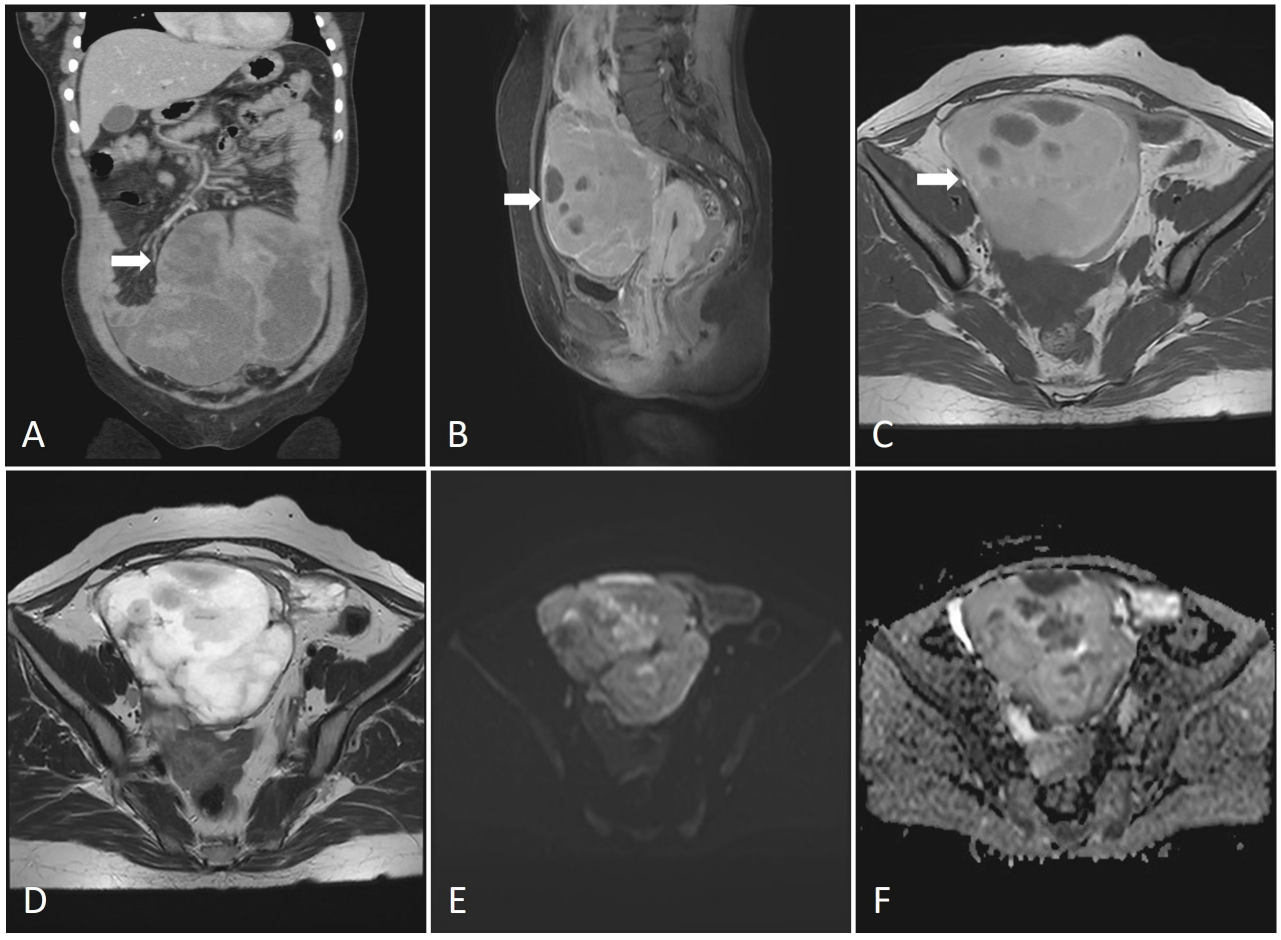
**(A)** CT imaging of the lesions (coronal plane).The abdominal mass was huge with inhomogeneous density and uneven enhancement. **(B)** MRI imaging of the lesions (sagittal plane). The MRI revealed a large cystic and solid lesion in the pelvic and abdominal cavity, internally segmented by multiple septa, with approximate dimensions of 11.7cm x 8.7cm x 19.1cm. **(C)** T1-weighted image (T1WI). The T1-weighted image (T1WI) showed heterogeneous slightly high signal intensity, accompanied by patchy areas of low signal intensity. **(D)** T2-weighted image (T2WI). The T2-weighted image (T2WI) exhibited heterogeneous slightly high signal intensity, with patchy low signal areas. **(E)** Diffusion-weighted imaging (DWI). Diffusion-weighted imaging (DWI) revealed patchy areas of high signal intensity. **(F)** Apparent diffusion coefficient (ADC). The apparent diffusion coefficient (ADC) demonstrated regions of low signal intensity.

The consent for publication was obtained from the patient and approved by Jinhua Central Hospital Medical Ethics Review Committee (2023No.202). In March 2021, a 47-year-old female patient was admitted to our hospital due to intermittent abdominal pain for a week. She has a height of 150 cm and a weight of 52 kg, resulting in a BMI of 22.507. The laboratory parameters is shown in **Supplementary Table 1**. A computed tomography (CT) scan revealed the presence of an abdominal mass (**Figure 1A**). The magnetic resonance imaging (MRI) revealed a large cystic and solid lesion in the pelvic and abdominal cavity, internally segmented by multiple septa, with approximate dimensions of 11.7cm x 8.7cm x 19.1cm (**Figure 1B**). The T1-weighted image (T1WI) showed heterogeneous slightly high signal intensity, accompanied by patchy areas of low signal intensity (**Figure 1C**). Meanwhile, the T2-weighted image (T2WI) exhibited heterogeneous slightly high signal intensity, with patchy low signal areas (**Figure 1D**). Diffusion-weighted imaging(DWI) revealed patchy areas of high signal intensity (**Figure 1E**), while the apparent diffusion coefficient(ADC) demonstrated regions of low signal intensity (**Figure 1F**). Post-contrast images showed a thickened and segmented wall with enhanced cystic regions, indicating a significant cystic-solid lesion in the pelvic and abdominal cavity originating from the right ovary. This is suggestive of a hemorrhagic cystadenoma. Based on these findings, a laparoscopic exploratory surgery was conducted, revealing a large, heterogeneous abdominal mass with a blackish-grey coloration. The mass, which had a soft consistency, exhibited significant fixation. Internal hemorrhage was observed within the cyst, and its size and extent of the mass were consistent with preoperative imaging findings. The mass extended from the abdominal aorta to the pelvis, with its upper portion reaching up to the posterior duodenal wall at the twelfth level. Complete timeline of the events is shown in **Supplementary Figure 1**.

Considering the presence of the tumor, an open abdominal surgery was necessary for the treatment, thus requiring a conversion to an open procedure. In collaboration with the hepatobiliary surgeon, a comprehensive investigation of the suspected duodenal tumor, which appeared to have a broad base, was conducted to ensure complete excision. Utmost caution was taken to prevent any inadvertent injury to both the inferior mesenteric artery and superior mesenteric vein. The rapid intraoperative pathology findings suggested the possibility of extra-gastric intestinal mesothelioma (clear cell sarcoma, pending confirmation), with the tissue type to be further validated through routine histopathological examination and immunohistochemical staining.

**Figure 2:**
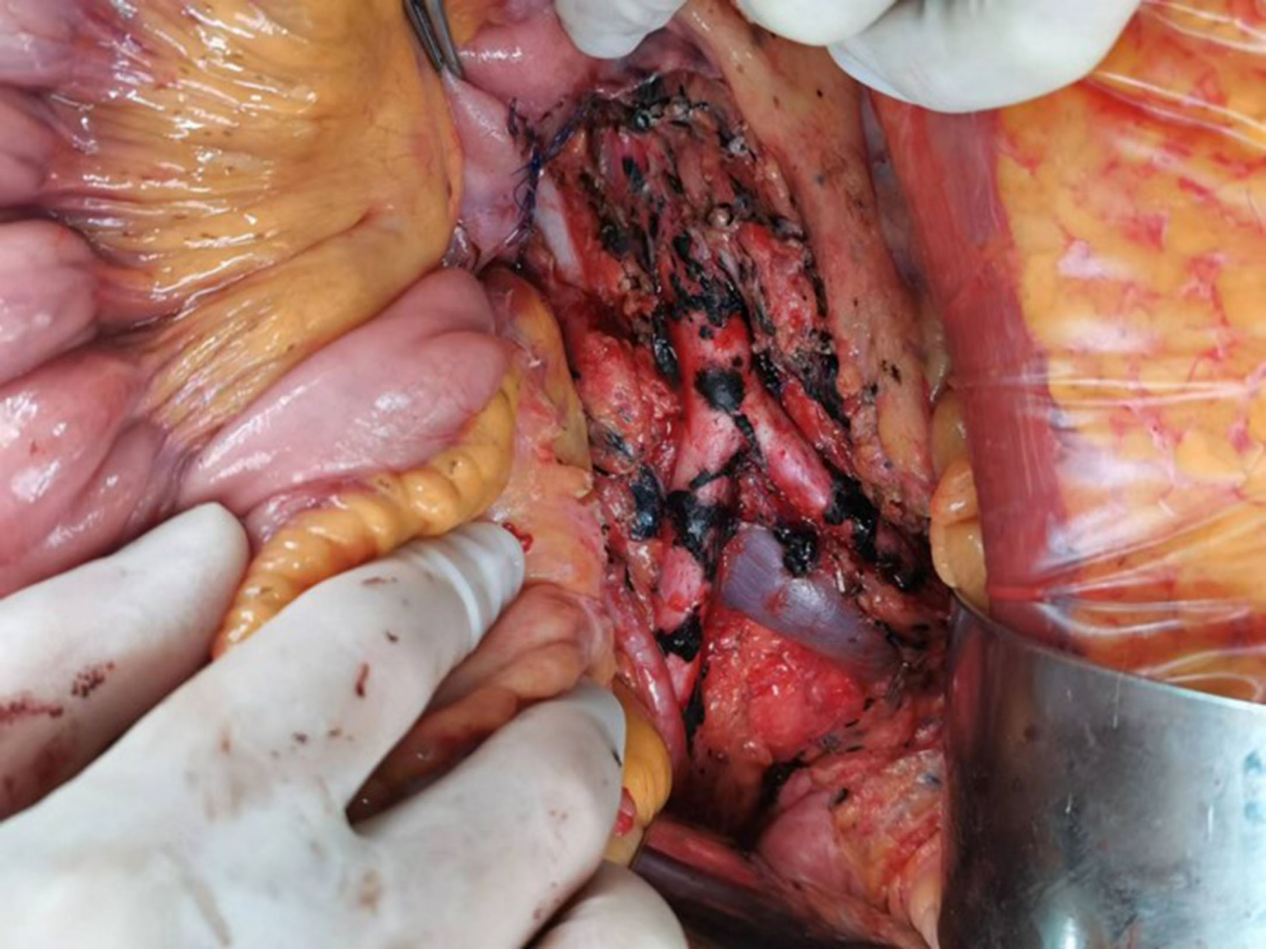
Postoperative photograph of the resected tumor. Lymphatic tissue encompassing the abdominal aorta, iliac arteries, and sacral region was resected along with excision of the greater omentum. To avoid catastrophic risks, the tumor capsule adhering to the surface of the abdominal aorta and bilateral iliac arteries was not resected.

**Figure 3:**
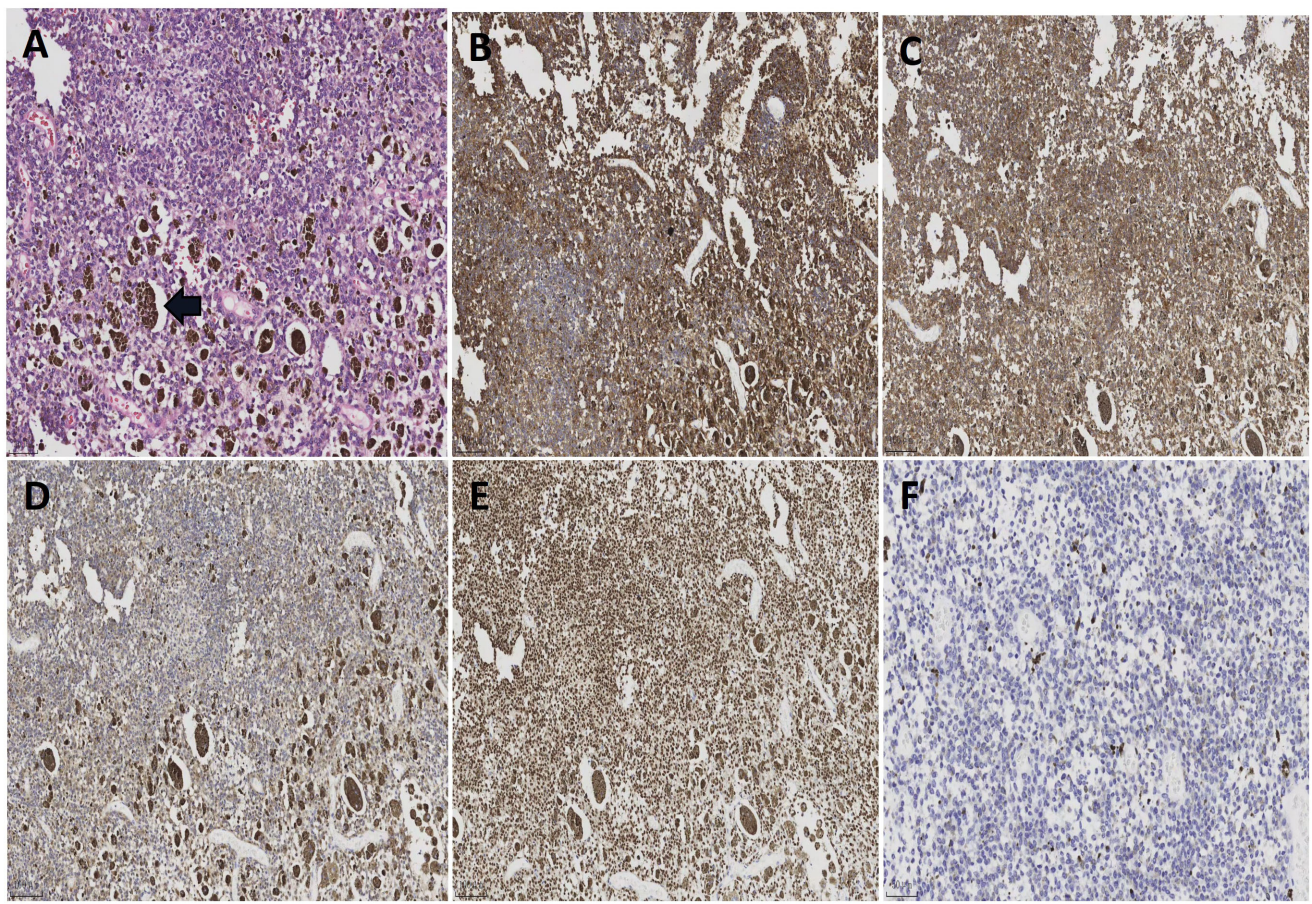
**(A)** Histological sections of patient’s tumor [H&E; ×200]. The tumor cells are oval in shape, with obvious nucleoli and significant melanin granule deposition. **(B)** Positive immunoactivity for MelanA (×100). **(C)** Positive immunoactivity for HMB45 (×100). **(D)** Positive immunoactivity for S-100 (×100). **(E)** Positive immunoactivity for SOX10 (×100). **(F)** Ki67 immunostain with a proliferative index of 2% (×200).

During the operation, further dissection of the retroperitoneal space surrounding the abdominal aorta revealed tumor infiltration into the walls of the abdominal aorta and bilateral iliac arteries. Subsequently, lymphatic tissue encompassing the abdominal aorta, iliac arteries, and sacral region was resected along with excision of the greater omentum. To avoid catastrophic risks, the tumor capsule adhering to the surface of the abdominal aorta and bilateral iliac arteries was not resected (**Figure 2**). The surgery has been completed, and a retroperitoneal drainage tube was inserted. The postoperative recovery went smoothly, and the surgical wounds healed well. The patient was discharged from the hospital 7 days after the surgery. Postoperative pathology revealed a MS(retroperitoneal mass, retroperitoneal lymphoid tissue, omentum). The reticular fiber staining analysis was positive. Immunohistochemical analysis results supported the diagnosis of MS (**Figure 3**). For example, HMB45, Melan-A, SOX10, S-100, and CD117 showed positive results. Ki67 expression was detected at 2% positivity. Desmin staining showed negative results. Follow-up examination is recommended based on these findings. After consultation with the Pathology Department of Fudan University Shanghai Cancer Center, it was still considered as MS. Molecular testing showed no definite mutation in exon 15 of the *BRAF* gene. Regular follow-up was conducted for 3 years after surgery, and there is no obvious recurrence and metastasis on MRI and PET-CT monitoring until now.

## Discussion

MS was first described by Millar in 1932 as malignant melanotic tumor of ganglion cells and subsequently termed as MS by Fu et al., in 1975 (1, 2). MS was previously classified as a benign tumor in the 2013 WHO classification, but in 2020 WHO classification, the term “MS” was revised to “malignant melanotic nerve sheath tumor (MMNST)” due to its malignant properties (3). Until now, the MMNST pathogenesis remains unclear. There are two different types of MS: the sporadic and the psammomatous melanotic schwannomas of Carney complex (4–9). Among them, up to 50% of these tumors are related to Carney complex, an autosomal dominant genetic syndrome characterized by spotty pigmentation of the skin, heart, breast, and uterine myxomas, primary pigmented nodular adrenocortical disease with Cushing’s syndrome, growth hormone-producing pituitary adenoma, testis/ovaries Sertoli cell tumors, thyroid adenomas, and breast adenomas (10, 11). In this case, the MS is located in the retroperitoneum, which is a rare site for this type of tumor. However, there is no indication of genetic inheritance or Carney syndrome, and no genetic mutations were found. The clinical symptoms of MS are non-specific, making preoperative diagnosis difficult. Preoperative diagnosis is based on MRI, which showed MS exhibit high signal intensity on T1WI and low signal intensity on T2WI compared to the non-schwannomas types which showed low signal intensity on T1 and high signal intensity on T2 sequences (12–15). The definitive diagnosis relies on pathological examination. MMNST typically expresses strong S100 and melanocyte markers such as SOX10, HMB45, Melan-A, and tyrosinase (16). The mitotic rate is the only histological feature predictive of clinical outcome. Study showed that a mitotic rate of more than 2/10 HPF would be associated with metastases (P=0.008) (17). MS are usually solitary, partially circumscribed or encapsulated, and heavily pigmented (18). Their diameters range from 0.5 cm to 25 cm, but most of them are over 5 cm (19).

MS exhibited potentially malignant biological properties, with the possibility of local recurrence and distant metastasis. Its prognosis is unpredictable. Torres-Mora et al. (16) reported MMNST local recurrence and metastatic rates was 35% and 44%, respectively (with an average follow-up of 55 months), and 73% of metastases occurred within four years. Metastases are commonly found in the lung and pleura. Currently, surgery is the primary treatment for MS. Due to the rarity of this tumor, there are no formal guidelines for adjuvant therapies, including radiotherapy and chemotherapy, and their use remains controversial (20, 21). However, it was reported in the literature that postoperative radiotherapy was used as an adjunctive treatment to reduce the possibility of local recurrence. A recent report emphasized the importance of adjuvant radiotherapy, in which the surgical exploration did not include sufficient surgical margins and hence 60 gray of adjuvant radiotherapy was taken. After 24 months follow-up, the patient showed no signs of recurrence or metastasis (22). In terms of chemotherapy, only Italian et al. (23) reported in 2011 that the application of chemotherapeutic agents could slow down the growth of tumor. Although the therapeutic efficacy of radiotherapy and chemotherapy has not been definitively confirmed, if it was proven, these treatments are recommended because of the aggressive potential of the lesions (24, 25). Other treatments such as targeted therapy and immune checkpoint therapy are currently emerging as promising options for tumor treatment (26). Thus, the use of immune checkpoint inhibitors in MMNST are limited to three reported cases. However, they provide some evidence suggesting that these inhibitors can offer symptomatic improvement and clinical response in some PD-L1-positive MMNST patients (18).

The pathophysiology and prognosis of MMNST require further data for a comprehensive understanding, as well as to improve survival rates. Surgical intervention should aim to achieve complete resection of MMNST to prevent the potential recurrence or transformation into more aggressive malignancies. In this case, the postoperative cytomorphological and immunohistochemical staining results indicated a low risk of malignancy, while the PET-CT assessment conducted two months after surgery revealed inactive metabolism in residual tissue. Consequently, adjuvant treatment was not administered to the patient who was advised regular follow-up and observation instead. Over a three-year period, the patient underwent annual PET-CT scans without any signs of recurrence or metastasis. However, there remains a residual tumor capsule on the surface of the blood vessels requiring long-term follow-up observation. If necessary, treatments such as chemotherapy, radiation therapy, or surgery should be considered.

## Conclusion

Retroperitoneal melanotic schwannoma is extremely rare, and currently, there are no reliable biomarkers for monitoring. Preoperative diagnosis is difficult and requires pathological and immunohistochemical confirmation. Surgery is the corerstone of MMNST treatment. There are no standard treatment guidelines for postoperative management. Therefore, we recommended thorough surgical resection, while taking care to protect of surrounding anatomical structures during the operation, as an effective treatment to avoid local tumor recurrence and preserve function. Furthermore, close long-term postoperative follow-up is necessary to accumulate diagnostic and treatment experience for better management of such cases.

## Data Availability

The raw data supporting the conclusions of this article will be made available by the authors, without undue reservation.
