# Supplementary material for: Rare giant retroperitoneal melanotic schwannoma: a case report and literature review
Source: Front Oncol. 2024 Aug 29;14:1448112. doi: 10.3389/fonc.2024.1448112 (PMC11390348; doi:10.3389/fonc.2024.1448112)
Supplement: Supplementary file 1 [file DataSheet1.docx]

Supplemental table 1 Laboratory parameters before and after surgery.

| Lab value | Reference value | Pre-operation | Post-operation | | |
| --- | --- | --- | --- | --- | --- |
|  |  |  | Day 1 | Day 3 | Day 6 |
| WBC | 3.5-9.5×10 ^9^/L | 6.48 | 11.76 | 9.65 | 6.52 |
| Neut | 40-75% | 75.7 | 87.9 | 87.7 | 71.8 |
| Hb | 115-150g/L | 104 | 86 | 88 | 116 |
| Plt | 125-350×10 ^9^/L | 223 | 395 | 279 | 373 |
| CRP | ＜8mg/L | 59.4 | 82.4 | 94.4 | 23.85 |
| AST | 13-35IU/L | 19.8 | 20 | 13 | 15 |
| ALT | 7-40IU/L | 10.7 | 6 | 12 | 13 |
| Cr | 40-135umol/L | 52 | 49.4 | 41.7 | 44.2 |

WBC, white blood cells; Neut, neutrophils; Hb, hemaglobin; Plt, platelets; CRP, C-reactive protein; AST, aspartate transaminase; ALT, alanaine transaminase; Cr, creatinine.


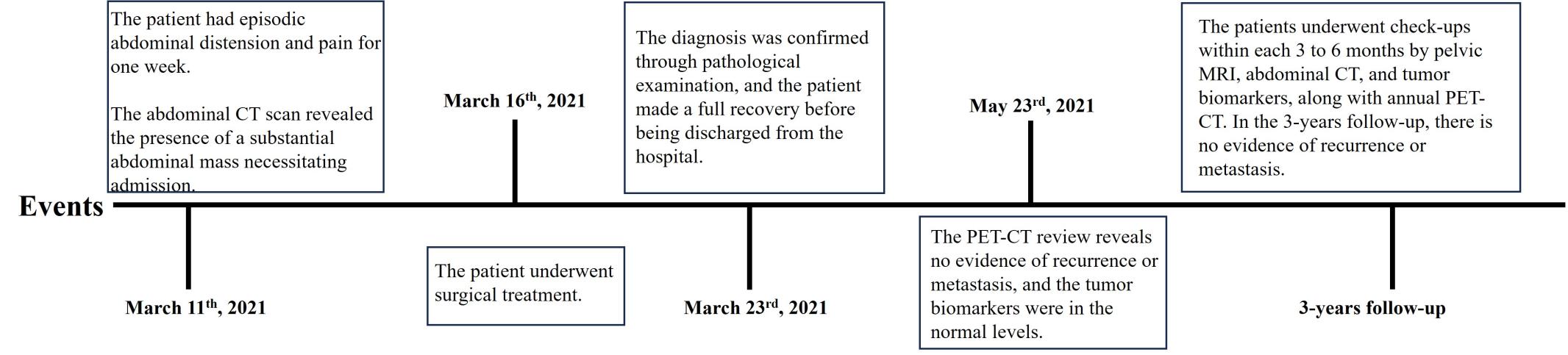


Supplemental figure 1 Timeline of events
